# Dermatitis artefacta in a 60 year old man: a case report

**DOI:** 10.1192/j.eurpsy.2024.1723

**Published:** 2024-08-27

**Authors:** P. Setién Preciados, C. Díaz Mayoral, E. Arroyo Sánchez

**Affiliations:** Servicio de Psiquiatría, Hospital Universitario Príncipe de Asturias, Alcalá de Henares, Spain

## Abstract

**Introduction:**

Dermatitis artefacta (DA), also known as factitial dermatitis, is a condition among factitious disorders, whereby self-induced skin damage is the means used to satisfy a conscious or unconscious desire to assume the sick role, particularly in those with an underlying psychiatric diagnosis or external stress. DA should be distinguished from malingering, in which skin damage may be inflicted for the purpose of secondary gain.

**Objectives:**

Review what dermatitis artefacta and factitious disorders in general consist of and the challenges they present.

**Methods:**

Presentation of a patient’s case and review of existing literature, in regards to factitial dermatitis and factitious disorders.

**Results:**

In general, in regards to factitious disorders in literature, the majority of patients were female with mean age at presentation at thirty. A healthcare or laboratory profession was reported most frequently, as well as a current or past diagnosis of depression was described more frequently than personality disorder in cases reporting psychiatric comorbidity, and more patients elected to self-induce illness or injury than simulate or falsely report it. Patients were most likely to present with endocrinological, cardiological and dermatological problems. In our patient’s case, common factors described previously are dermatological lesions, comorbid psychiatric disorder and the beginning of the disorder at an earlier age.

Specifically, when it comes to DA, the hallmarks of diagnosis include self-inflicted lesions in accessible areas of the face and extremities that do not correlate with organic disease patterns. Importantly, patients are unable to take ownership of the cutaneous signs.

Management in these cases is challenging, and different modalities may be employed, including topical therapies, oral medications, and cognitive behavioural therapy; adopting a multidisciplinary team approach has been shown to be beneficial in allowing patients to come to terms with their illness in an open, non judgmental environment.

**Conclusions:**

DA is a rare cutaneous condition that must be considered when the clinical presentation is atypical and investigations do not yield an alternate diagnosis. Few are referred to psychiatric services and even fewer accept care. They have a protracted course, complicated by repeated hospitalizations, ultimately leading to their premature deaths. Clear guidelines on the management of these patients need to be set to protect both patients and providers in light of the ethical and legal considerations.

**Disclosure of Interest:**

None Declared

